# Influence of creatine pyruvate on newly received cattle: insights from metagenomics and metabolomics

**DOI:** 10.1186/s12866-025-04384-8

**Published:** 2025-10-10

**Authors:** Kang Mao, Guwei Lu, Qinghua Qiu, Yitian Zang, Kehui Ouyang, Xianghui Zhao, Xiaozhen Song, Lanjiao Xu, Huan Liang, Mingren Qu, Yanjiao Li

**Affiliations:** College of Animal Science and Technology, Jiangxi Agricultural University, Nanchang, 330045 China

**Keywords:** Creatine pyruvate, Calves transportation stress, Rumen metagenomics, Rumen metabolomics, Serum metabolomics

## Abstract

Transport stress is a critical factor affecting the health and growth performance of beef cattle, potentially leading to oxidative stress, inflammation, and metabolic disorders. Creatine pyruvate (CrPyr), as a potential stress alleviator, has unclear mechanisms of action. We monitored the growth of 17 Simmental calves (control, *n* = 8; CrPyr, *n* = 9) over 30 days post-transportation, collecting rumen and blood samples on days 1/4, and 30. This study aims to investigate the effects of CrPyr on the growth performance, rumen microbiome, and metabolome of calves subjected to transport stress. Results showed that CrPyr increased average daily gain and antioxidant capacity, while reducing the level of stress hormones and inflammation. In the 4 days post-transport, CrPyr mainly increases *Ruminococcus* abundance to boost ruminal nitrogen metabolism, providing substrates for microbial protein synthesis. CrPyr also provides energy for the proliferation of *Ruminococcus* by regulating ATP synthesis genes (ATPVC) and enriching purine metabolism products. Meanwhile, it strengthens the host’s amino acid metabolism, especially aspartate, to enhance antioxidative capacity. By day 30, CrPyr primarily boosts *Prevotella* abundance to regulate VFA synthesis, supplying host energy. It regulates the ATP synthesis gene *ATPF0A* and enriches purine metabolism products, supporting *Prevotella* growth. Increased citric acid and ATP levels further aid host growth. The findings distinctly demonstrate that the mechanisms by which CrPyr alleviates transport stress through the regulation of the rumen microbiome and metabolome, and confirms that its effects are time-dependent. These findings provide a theoretical basis for the development of stress-alleviation strategies based on CrPyr and hold significant implications for enhancing the health and production performance of beef cattle.

**Supplementary Information:**

The online version contains supplementary material available at 10.1186/s12866-025-04384-8.

## Background

Transportation is an essential yet challenging aspect of the beef cattle industry, significantly impacting animal health and welfare [1]. During this process, cattle are subjected to a multitude of stressors, including varying handling practices, exposure to unfamiliar individuals, deprivation of food and water, and fluctuations in temperature [2, 3]. These stressors collectively pose a substantial threat to the health of beef cattle. Particularly under conditions of fasting and water restriction, cattle not only endure energy depletion, hunger, and hypoglycemia but also become more susceptible to pathogens [1, 4, 5]. Calves are especially vulnerable due to their limited body fat reserves and underdeveloped immune systems, making them less capable of resisting pathogenic invasions [4, 6]. Consequently, post-long-distance transportation, the growth performance of calves is often severely compromised, leading to substantial economic losses. For instance, Charolais bulls showed a progressive increase in weight loss from 4.7 to 7.5% after transportation durations ranging from 6 to 24 h [7]. Therefore, minimizing stress levels during and after transportation is a primary objective for the cattle industry.

Optimizing routine management practices for calves before and after transportation is a recognized method for reducing stress levels. For example, the addition of live yeast (LY), mannan oligosaccharides (MOS), and organic selenium (Se) can enhance the immune capacity and growth performance of newly received cattle [8]. Similarly, A 10% molasses supplement, an energy-based nutritional additive, can alter gastrointestinal barrier functions, thereby enhancing anti-inflammatory capabilities and growth performance in newly received cattle [9]. However, the underlying mechanisms remain unclear. Creatine pyruvate (CrPyr), as a new multifunctional nutrient comprised of 40% pyruvate and 60% creatine, both natural body intermediate metabolites [10], plays a significant role in rumen function. Pyruvate is an important fermentation intermediate in the rumen, mainly originating from the carbohydrate metabolism by rumen microorganisms [11]. The conversion of pyruvate to VFA generates adenosine triphosphate (ATP), thus promoting microbial growth. Simultaneously, creatine, a natural nitrogenous organic acid integral to energy and protein metabolism [12], serves as an important nitrogen source for microbial protein synthesis. Therefore, supplementing with CrPyr is an effective option under conditions of insufficient energy supply in newly received cattle.

The rumen’s ability to convert complex polysaccharides into VFAs, microbial proteins, and vitamins is essential for alleviating stress and maximizing feed efficiency [13]. A stable ruminal microecology is indispensable for the animal’s health, as minor disturbances can notably influence nutrient absorption and productivity [14]. Furthermore, disruptions in the rumen flora can escalate inflammatory responses, thereby affecting the host’s overall well-being [15]. Therefore, maintaining a stable and healthy rumen microbiome is imperative for the health and productivity of ruminants. A study on Xianan cattle highlighted marked changes in essential cellulolytic bacteria post-transport, potentially influencing the growth of the cattle [16]. This imbalance can give rise to various digestive complications and other health concerns. Thus, a pivotal strategy to optimize the growth performance of newly received cattle lies in fostering the recovery of rumen homeostasis during this critical transition period.

Our previous research has demonstrated that CrPyr has the potential to alleviate stress. Specifically, in beef cattle treated with CrPyr, the increased generation of adenosine triphosphate (ATP) during fatty acid β-oxidation and the citrate cycle, along with the upregulation of microbial protein synthesis in the rumen, may help decrease oxidative stress, regulate energy metabolism, and improve rumen fermentation characteristics under heat stress [17, 18]. Additionally, CrPyr reduces serum cortisol and lipopolysaccharide (LPS) levels and promotes the restoration of the rumen microbiota, thereby mitigating transportation stress in beef cattle [19]. Therefore, we speculate that CrPyr likely exerts a growth-promoting effect on newly received cattle via its regulatory function in maintaining rumen homeostasis. However, it is not clear which microbial taxa and metabolic pathways mediate CrPyr to correct rumen flora disorders in newly received cattle, and how changed rumen flora and their metabolites induced by CrPyr affect the host growth and metabolism. This limits the application of CrPyr in stress relief in newly received cattle. This study aims to elucidate how CrPyr promotes the growth of newly received calves by regulating the rumen microbiota and metabolome, through analyses of rumen metagenomics, rumen metabolomics, and host metabolomics. The findings of this study will provide a theoretical basis for the development of CrPyr-based stress mitigation strategies.

## Result

### Growth performance

**Fig. 1:**
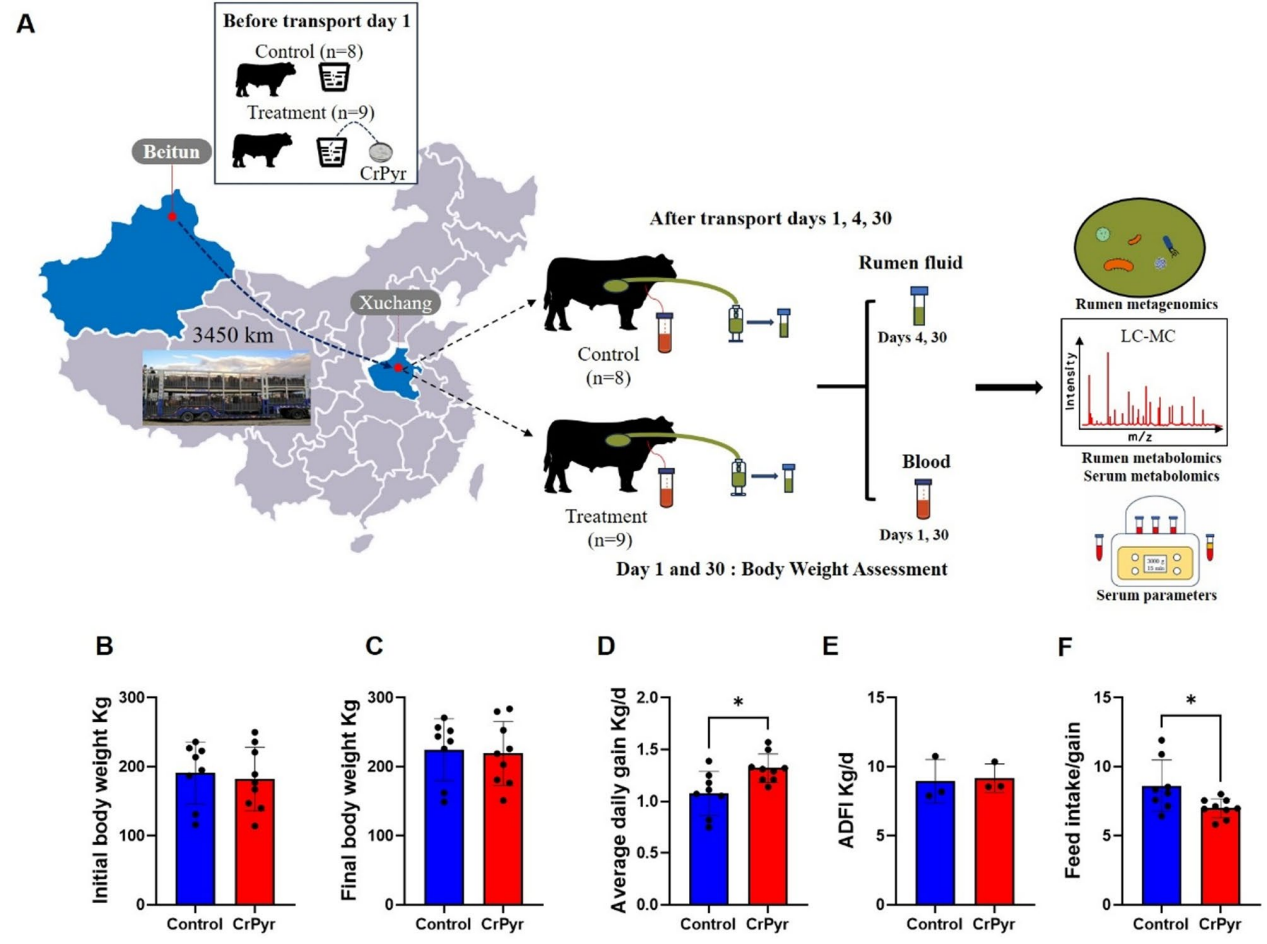
The flowchart of this study and basic indicators. **A** Study and sampling design of the trial. **B-F** Growth of performance of newly received cattle during 30 days after arrival. *P < 0.05, ** *P* < 0.01

The schematic representation of the experimental design is shown in Fig. 1 A. The experimental cattle were weighed individually on the first and last day. As shown in Fig. 1B-D, there were no significant differences in initial body weight, final body weight, and average daily feed intake (ADFI) of newly received cattle between the CrPyr and control groups (*P* > 0.05). However, the average daily gain (ADG) of newly received cattle was higher in the CrPyr group than in the control group (*P* < 0.05). The CrPyr group had a lower feed intake/gain (F: G) value compared to the control group (Fig. 1 F; *P* < 0.05).

### Serum parameters

**Fig. 2:**
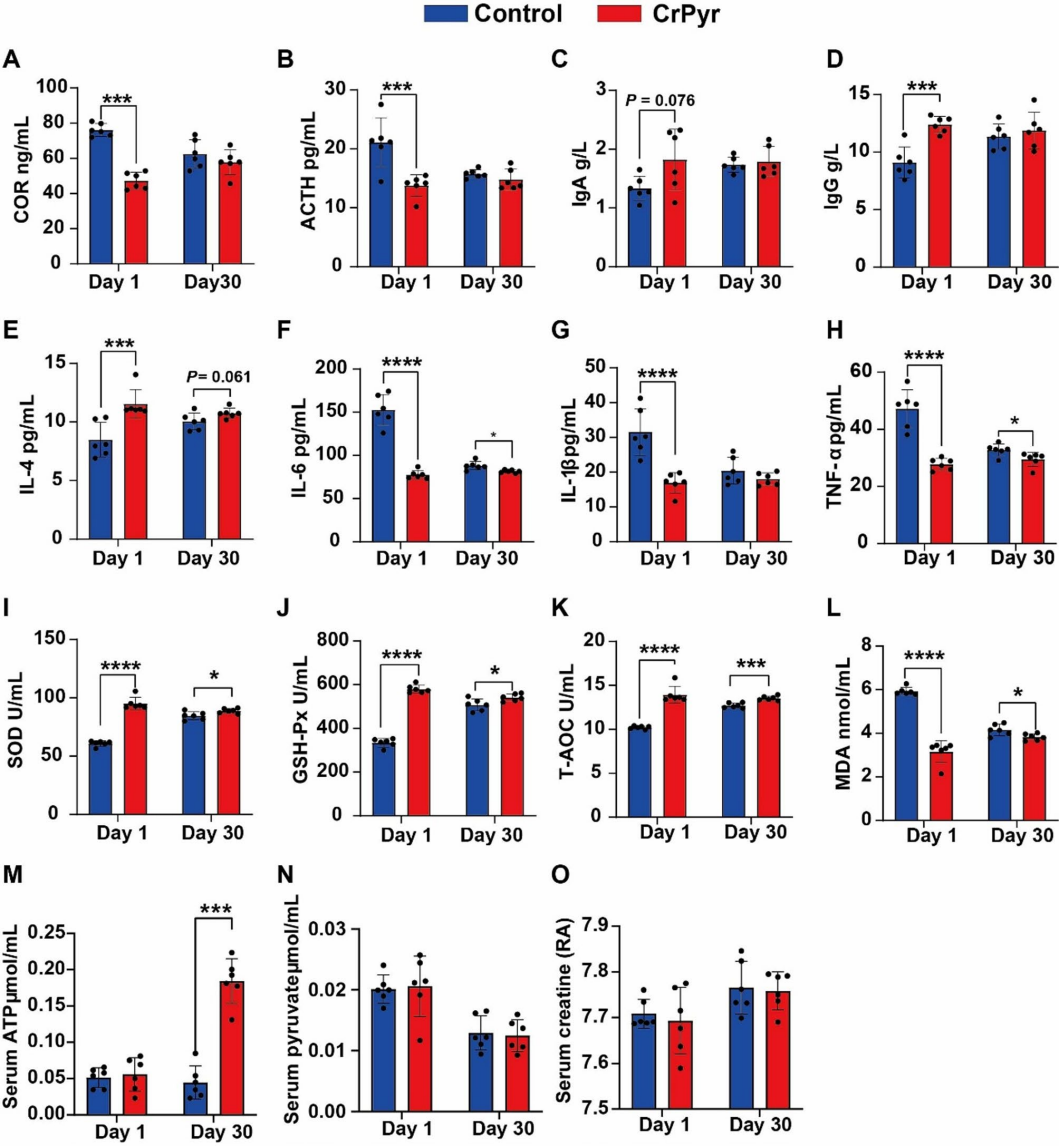
Effects of CrPyr on serum parameters of newly received cattle. **A-D** Hormone levels and immune indicators. **E-H** Levels of inflammatory factors. **I-L** Antioxidant capacity. **M-O** Energy metabolism related products. Day 1 = on day 1 after transport; Day 30 = at day 30 after transport. *P < 0.05, **P < 0.01, ***P < 0.001, ****P < 0.001. ACTH = adreno cortico tropic hormone; COR = cortisol; IL-1β = interleukin-1β; IL-4 = interleukin-4; TNF-α = tumor necrosis factor-α; T-AOC = total antioxidant capacity; SOD = superoxide dismutase; GSH-PX = glutathione peroxidase; MDA = malondialdehyde. RA = Relative abundance

Upon arrival, significant differences were observed between the CrPyr-supplemented group and the Control group in various physiological parameters. Notably, on day 1 after arrival, the CrPyr group exhibited significantly reduced levels of stress indicators, adrenocorticotropic hormone (ACTH) and cortisol (COR) (Fig. 2A, B; *P* < 0.05). This suggests that CrPyr may alleviate stress responses in newly received cattle. Conversely, the CrPyr group demonstrated enhanced immune function, evidenced by significantly elevated levels of immunoglobulins A (IgA) and G (IgG) (Fig. 2C, D; *P* < 0.05). These findings indicate that CrPyr supplementation could potentially bolster the immune system in cattle undergoing transportation stress. Furthermore, the CrPyr group showed a marked increase in the anti-inflammatory cytokine interleukin-4 (IL-4) (Fig. 2E, *P* < 0.05), This elevation in IL-4 suggests that CrPyr may promote anti-inflammatory responses. In contrast, the CrPyr group had significantly lower levels of pro-inflammatory factors, including interleukin-6 (IL-6), interleukin-1β (IL-1β), and tumor necrosis factor-α (TNF-α), compared to the Control group (Fig. 2F-H, *P* < 0.05). These results collectively imply that CrPyr supplementation may modulate inflammatory responses in newly introduced cattle, potentially mitigating the negative effects of transportation stress on inflammation and overall health. On day 30 post-arrival, the CrPyr group continued to show significantly reduced levels of the pro-inflammatory IL-6 and TNF-α compared to the control group at the same time point (Fig. 2F, H; *P* < 0.05). This sustained reduction in inflammatory markers suggests a prolonged anti-inflammatory effect of CrPyr supplementation. In terms of antioxidant status, the CrPyr group exhibited significantly elevated serum levels of superoxide dismutase (SOD), glutathione peroxidase (GSH-Px), and total antioxidant capacity (T-AOC) on both day 1 and day 30 post-arrival (Fig. 2 I-K, *P* < 0.05). Concurrently, the level of the lipid peroxidation product malondialdehyde (MDA) was significantly lower in the CrPyr group (Fig. 2L, *P* < 0.05). These findings indicate that CrPyr supplementation enhances the antioxidant defense system, potentially mitigating oxidative stress in cattle following transportation. Regarding energy metabolism, no significant changes were observed in the levels of adenosine triphosphate (ATP), pyruvate, and creatine on day 1 post-transport. However, by day 30 post-transport, the CrPyr group demonstrated significantly higher ATP levels compared to the control group (Fig. 2 M-O, *P* < 0.05). This increase in ATP suggests that CrPyr supplementation may support energy metabolism and promote recovery in cattle after a prolonged period of transportation stress.

### Profiling of the rumen metagenome

Metagenome sequencing generated a total of 1,862,911,304 reads, with 77,624,054.33 ± 1,201,522.54 reads (mean ± standard error of the mean [SEM]) per sample (Table S1-2). After quality control and removing host genes, a total of 1,817,868,612 reads were retained, with 75,744,525.50 ± 1,204,579.70 per sample. After de novo assembly, a total of 19,528,591 contigs were generated (the N50 length of 759.50 ± 13.72 bp), with 813,691 ± 25,051.69 per sample. The rumen metagenome consisted of 97.60% bacteria (785,959,696 sequences), 1.19% archaea (9,617,494 sequences), 0.80% eukaryotes (6,409,436 sequences), and 0.27% viruses (2,155,903 sequences; Table S3). Based on species level, our analysis did not reveal significant differences in the Shannon and Chao1 diversity indices between the two groups, indicating similar microbial diversity. Furthermore, principal component analysis (PCA) based on the Bray-Curtis dissimilarity index demonstrated no significant variations in the overall microbial composition between the groups on both day 4 and day 30 (Fig. S1).

### Rumen Microbiome composition as determined by metagenomics

**Fig. 3:**
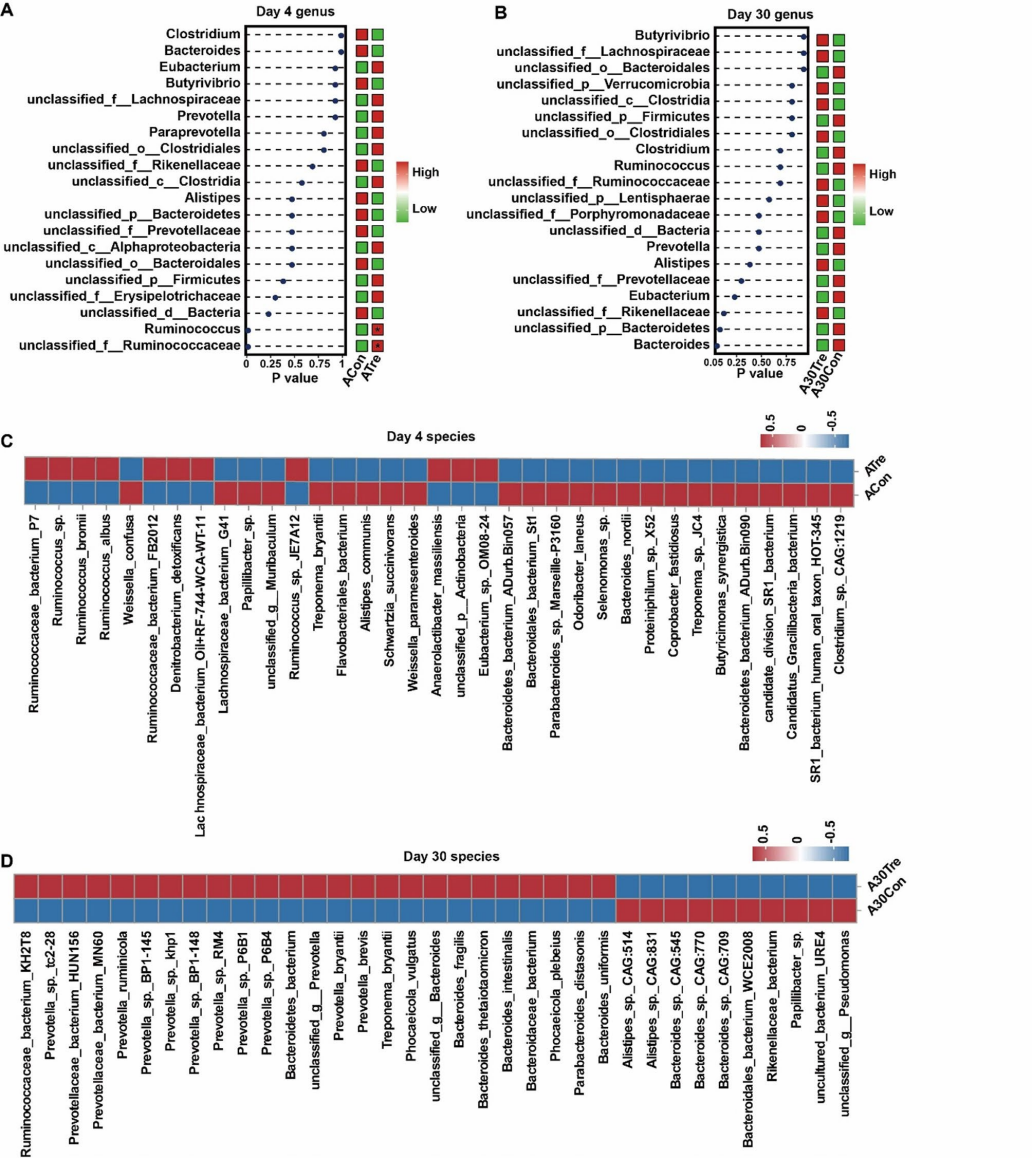
Microbial compositional profiles of newly received cattle at days 4 and 30 after transport. **A-B** the analysis of differences in the top 20 species at the genus level. **C-D** the analysis of differences in species with relative abundance > 0.01% at the species level. The top 35 different species are displayed. Differences at both the genus and species levels were analyzed using the Wilcoxon rank-sum test. *, *P* < 0.05 Day 1 = on day 1 after transport (ACon: Control; ATre: CrPyr); Day 30 = at day 30 after transport (A30Con: Control; A30Tre: CrPyr)

The dominant bacterial phyla included Bacteroidetes and Firmicutes, which accounted almost 80% of the bacteria, while no significant differences in the Bacteroidetes and Firmicutes between the two groups were observed (Fig. S2C, D; *P* > 0.05). At the genus level, differential analysis was conducted on the top 20 most abundant bacterial genera. The results indicated a significant increase in the relative abundance of *unclassified_f__Ruminococcaceae* and *Ruminococcus* in the CrPyr group on day 4 (Fig. 3A; *P* < 0.05). However, no significant differences in bacterial genera were observed on day 30 between the two groups (Fig. 3B). At the species level, we performed rank-sum tests on bacteria with relative abundances in the top 0.01%. The analysis identified 35 and 132 significantly differentially abundant species on days 4 and 30, respectively (Table. S4). Due to the large number of differential bacteria, we selected the top 35 by relative abundance for visualization (Fig. 3C, D; *P* < 0.05). On day 4, among the 11 species with significantly increased abundance in the CrPyr group, 6 belonged to the genus *Ruminococcus*, including *R. bacterium P7*, *R. sp*., *R. bromii*, *R. albus*, *R. bacterium FB2012*, and *R. sp. JE7A12* (Fig. 3C). The response to CrPyr supplementation highlights a significant impact on *Ruminococcus* genus. Progressing to day 30, the CrPyr group showed a significant upregulation of 25 bacterial species, among which 10 were *Prevotella* species, including *P. sp. tc2-28*, *P. ruminicola*, *P. sp. BP1-145*, *P. sp. khp1*, *P. sp. BP1-148*, *P. RM4*, *P. sp. P6B1*, *P. sp. P6B4*, *P. bryantii*, and *P. brevis* (Fig. 3D). This shift towards *Prevotella* species suggests a dynamic change in the microbial community composition over time in response to CrPyr supplementation.

### Rumen microbial functions as determined by metagenomics

**Fig. 4:**
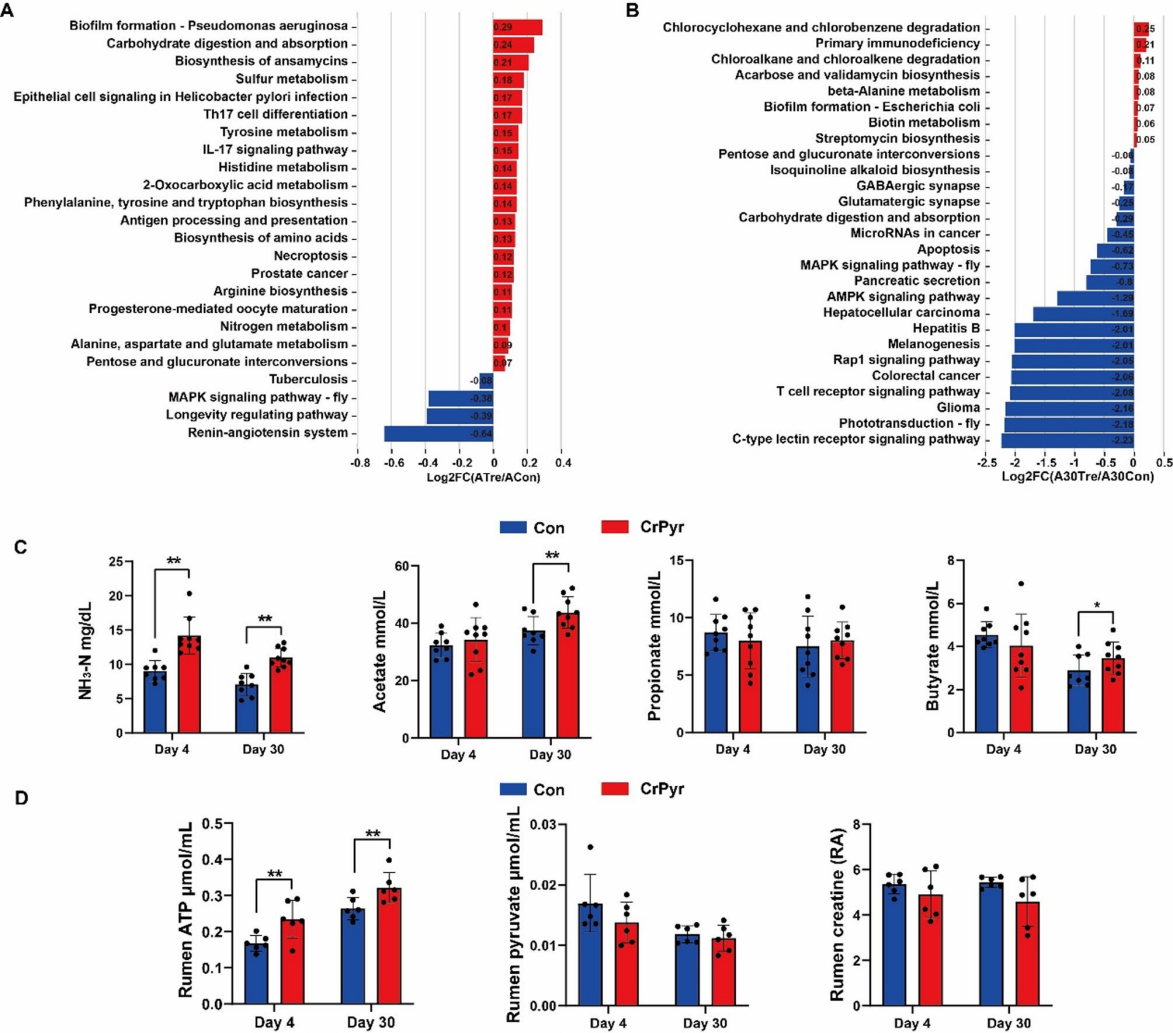
Differential rumen KEGG Function and rumen fermentation characteristics of newly received cattle at days 4 and 30 after transport. **A-B** CrPyr/Con fold changes of significantly enriched metabolic pathways. **C** Rumen fermentation characteristics. **D** Rumen energy metabolism related products. The KEGG function were analyzed using the Wilcoxon rank-sum test. Rumen fermentation characteristics and energy metabolism related products were analyzed using the *t* test. *, *P* < 0.05; **P < 0.01. Day 1 = on day 1 after transport (ACon: Control; ATre: CrPyr); Day 30 = at day 30 after transport (A30Con: Control; A30Tre: CrPyr)

To investigate the functional differences in the rumen microbiome between the control and CrPyr groups at two distinct time points, we conducted a KEGG (Kyoto Encyclopedia of Genes and Genomes) enrichment analysis using metagenomic data. For the KEGG profiles, we applied a screening criterion of RPKM (Reads Per Kilobase of transcript, per Million mapped reads) greater than 100. As a result, A total of 214 and 322 third-level KEGG pathways were identified on days 4 and 30, respectively (Table S5). The significantly differential KEGG pathways on two time points are shown in Fig. 4A, B. On day 4, there were 20 pathways enriched in ATre group, and 4 pathways enriched in ACon group. Most of the differential pathways were associated with nitrogen metabolism, especially amino acid metabolism. Specifically, nitrogen metabolism (*P* = 0.008), Tyrosine metabolism (*P* = 0.04), Histidine metabolism (*P* = 0.01), Arginine biosynthesis (*P* = 0.01) (Fig. 4A). On day 30, a total of 27 significantly different metabolic pathways were identified (Fig. 4B). Among these, 8 pathways were related to metabolism, including Chlorocyclohexane and chlorobenzene degradation, Chloroalkane and chloroalkene degradation, Acarbose and validamycin biosynthesis, beta-Alanine metabolism, Biotin metabolism, Streptomycin biosynthesis, Pentose and glucuronate interconversions, and Isoquinoline alkaloid biosynthesis. The remaining 19 pathways were associated with Human Diseases, Cellular Processes, Organismal Systems, and Environmental Information Processing. Since these pathways have been less studied in the context of rumen fermentation systems, they are not discussed in detail in this study.

**Fig. 5:**
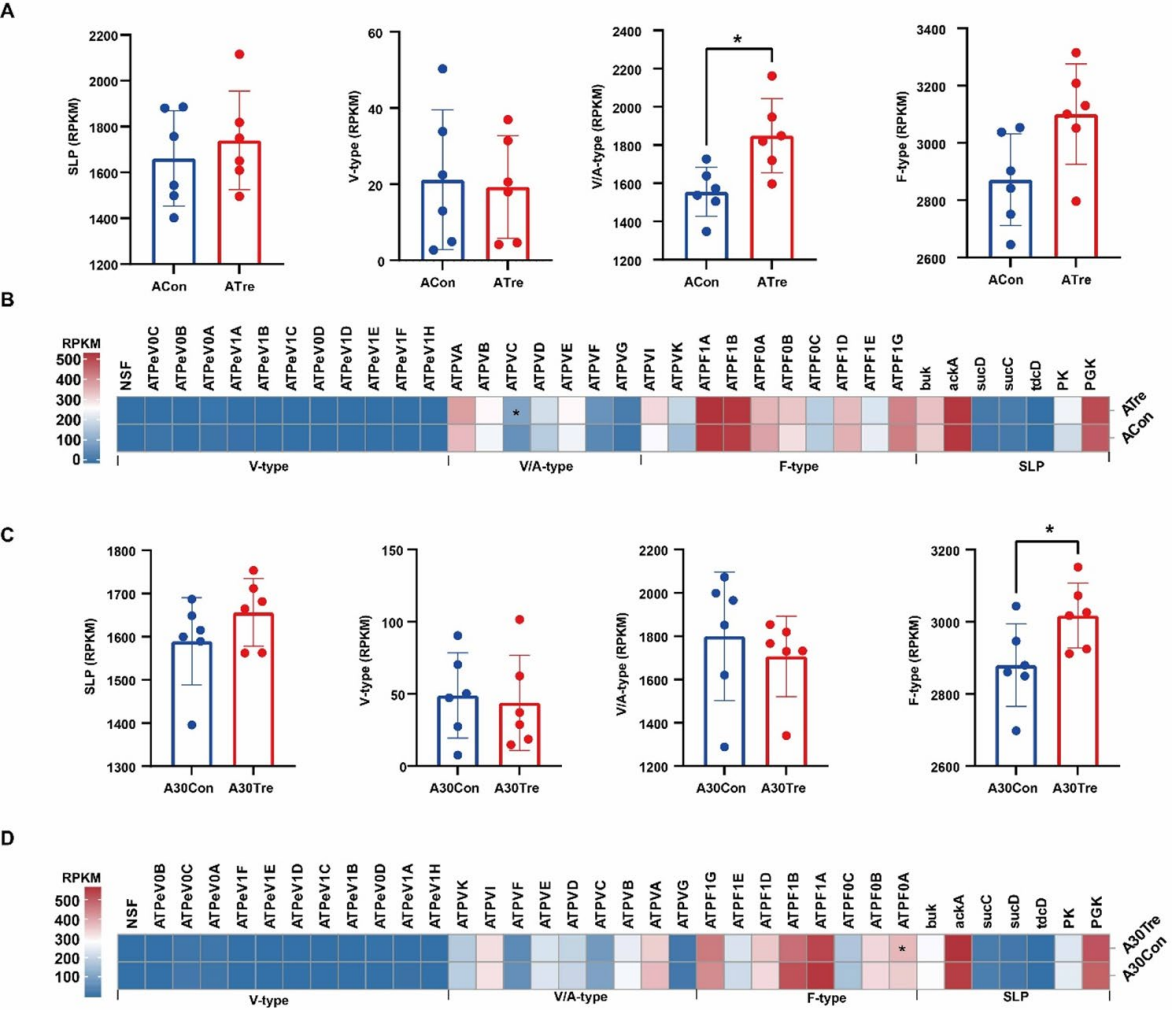
Effects of CrPyr on ATP synthesis-related genes in newly received cattle. **A-B** the effects on ruminal ATP synthesis in calves on day 4 after transport. **C-D** the effects on ruminal ATP synthesis on day 30 after transport. The ATP synthesis-related genes were analyzed using the Wilcoxon rank-sum test. *, *P* < 0.05; Day 1 = on day 1 after transport (ACon: Control; ATre: CrPyr); Day 30 = on day 30 after transport (A30Con: Control; A30Tre: CrPyr)

Additionally, our investigation into ruminal energy supply revealed significant changes in key metabolites and gene expression patterns. Specifically, the concentrations of NH_3_-N and ATP exhibited marked increases on both day 4 and day 30 (Fig. 4C, D). In contrast, while the concentrations of acetate and butyrate remained unchanged on day 4, they showed significant elevation by day 30. Given that ATP serves as the primary energy currency for microbial metabolism, and considering the involvement of CrPyr in energy supply, we conducted an in-depth analysis of genes associated with ATP synthesis. In this study, we identified a total of 36 microbial genes related to ATP production, including 7 genes associated with substrate-level phosphorylation (SLP) enzymes and 29 genes encoding ATP synthases (F-type, V/A-type, and V-type) (Table S6). Differential expression analysis of these genes revealed distinct temporal patterns in response to CrPyr treatment. Specifically, on day 4, CrPyr treatment significantly upregulated the expression of V/A-type ATP synthase genes, whereas on day 30, it significantly increased the expression of F-type ATP synthase genes (Fig. 5A, C; *P* < 0.05). Further examination of specific gene abundances showed that the abundance of *ATPVC* (V/A-type synthase gene) was significantly elevated on day 4, while the abundance of *ATPF0A* (F-type synthase gene) increased significantly on day 30 (Fig. 5B, D; *P* < 0.05). These findings suggest that CrPyr enhances ATP synthesis by modulating the expression and abundance of key ATP synthase genes. Consequently, this upregulation of ATP production likely supports increased energy availability for microbial metabolism and overall host energy supply.

### Rumen metabolome and serum metabolome

**Fig. 6:**
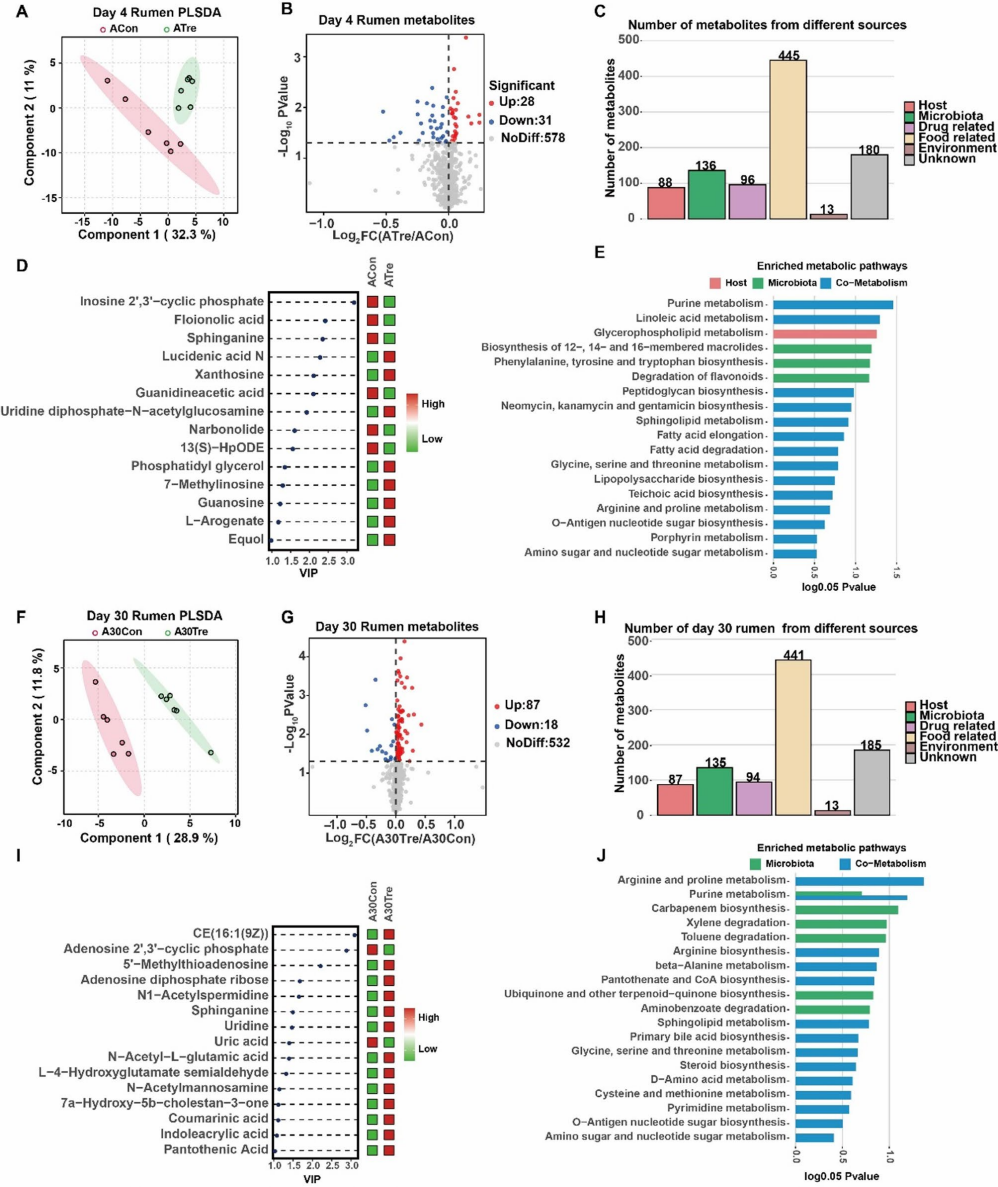
Rumen metabolites between CrPyr and Control at day 4 and 30. **A**,** F** the partial least squares discriminant (PLSDA) analysis of the rumen metabolome. **B**,** G** the volcano map of metabolites identified by the rumen metabolome. **C**,** H** show the number of metabolites from different sources. **D**,** I** show the differential metabolites from microbial and host origins. **E**,** J** show the metabolic pathway enrichment analysis according to different categories of metabolites. Day 1 = on day 1 after transport (ACon: Control; ATre: CrPyr); Day 30 = on day 30 after transport (A30Con: Control; A30Tre: CrPyr)

To further elucidate the metabolic changes induced by CrPyr treatment, we conducted ruminal metabolite profiling at two distinct time points (day 4 and day 30). Partial least squares-discriminant analysis (PLS-DA) revealed clear separation in the metabolic profiles between the CrPyr and Control groups, indicating distinct metabolic clusters (Fig. 6A, F). A total of 637 unique compounds were detected in the rumen fluid (Table S7). Among these, 59 and 105 metabolites were significantly different between the groups on day 4 and day 30, respectively (Fig. 6B, G, Table S8). We Further investigated the origins of these metabolites and found that on day 4, 88 metabolites were derived from the host, while 136 were of microbial origin (Fig. 6C). On day 30, the numbers were 87 and 135, respectively (Fig. 6H). Focusing on the significantly different metabolites originating from both the host and microbes, we identified specific changes in their abundance. On day 4, metabolites such as inosine 2’,3’-cyclic phosphate, floionolic acid, sphinganine, Guanidineacetic acid, narbonolide, and 13(S)-HpODE were significantly upregulated in the ACon group, whereas lucidenic acid N, xanthosine, uridine diphosphate-N-acetylglucosamine, phosphatidyl glycerol, 7-Methylinosine, guanosine, L-arogenate, and equol were significantly upregulated in the ATre group (Fig. 6D). These metabolites were primarily involved in pathways related to Purine metabolism, Linoleic acid metabolism, Glycerophospholipid metabolism, and Phenylalanine, tyrosine, and tryptophan biosynthesis (Fig. 6E). By day 30, the metabolic changes were more pronounced in the A30Tre group. Only adenosine 2’,3’-cyclic phosphate and uric acid were significantly upregulated in the A30Con group. In contrast, metabolites such as CE(16:1(9Z)), 5’-methylthioadenosine, adenosine diphosphate ribose, N1-acetylspermidine, sphinganine, uridine, N-Acetyl-L-glutamic acid, L-4-hydroxyglutamate semialdehyde, N-acetylmannosamine, 7a-hydroxy-5b-cholestan-3-one, coumarinic acid, indoleacrylic acid, and pantothenic acid were significantly upregulated in the A30Tre group (Fig. 6I). These metabolites were predominantly enriched in pathways related to arginine and proline metabolism, purine metabolism, and carbapenem biosynthesis (Fig. 6J).

**Fig. 7:**
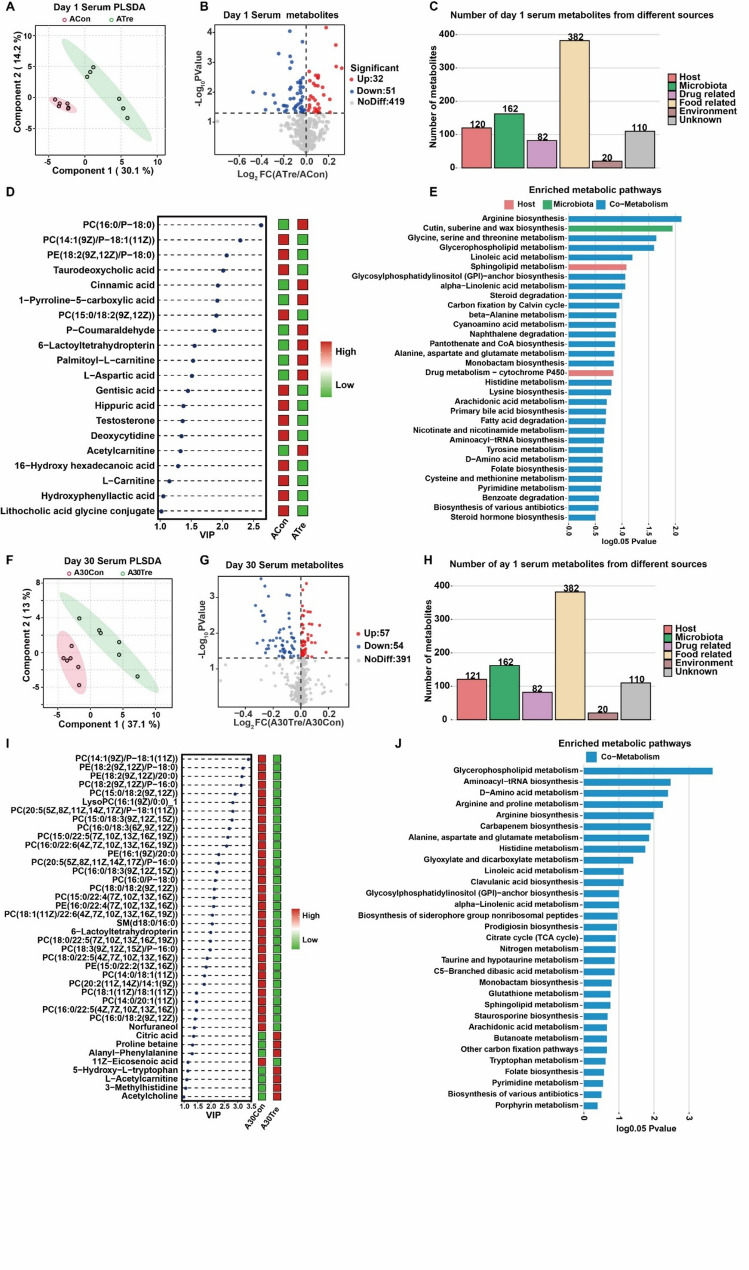
Rumen metabolites between CrPyr and Control at day 4 and 30. **A**,** F** the partial least squares discriminant (PLSDA) analysis of the rumen metabolome. **B**,** G** the volcano map of metabolites identified by the rumen metabolome. **C**,** H** show the number of metabolites from different sources. **D**,** I** show the differential metabolites from microbial and host origins. **E**,** J** show the metabolic pathway enrichment analysis according to different categories of metabolites. Day 1 = on day 1 after transport (ACon: Control; ATre: CrPyr); Day 30 = on day 30 after transport (A30Con: Control; A30Tre: CrPyr)

In the serum metabolome analysis, distinct clustering was observed between the two groups on days 1 and 30 post-arrival (Fig. 7A, F). A total of 502 compounds were identified in the serum metabolome (Table S9), with 83 and 111 metabolites significantly differing between the groups on days 1 and 30, respectively (Fig. 7B, G, Table S10). The origins of these metabolites were also investigated, showing 120 host-derived and 162 microbial-derived metabolites on day 1 (Fig. 7C), 121 and 162 on day 30 (Fig. 7H). Focusing on the significantly different metabolites from both host and microbial sources, we identified specific changes in their abundance. On day 1, PC(16:0/*P* − 18:0), cinnamic acid, 1 − pyrroline − 5−carboxylic acid, P − coumaraldehyde, 6 − lactoyltetrahydropterin, palmitoyl − L−carnitine, L − aspartic acid, and acetylcarnitine were enriched in the ATre group. In contrast, 12 metabolites were enriched in the ACon group, such as PC(14:1(9Z)/*P* − 18:1(11Z)), PE(18:2(9Z,12Z)/*P* − 18:0), taurodeoxycholic acid, PC(15:0/18:2(9Z,12Z)), gentisic acid, hippuric acid, testosterone, deoxycytidine, 16 − hydroxy hexadecanoic acid, L − carnitine, hydroxyphenyllactic acid, and lithocholic acid glycine conjugate (Fig. 7D; VIP > 1, *P* < 0.05). These metabolites were primarily involved in pathways related to Arginine biosynthesis, Cutin, suberine, and wax biosynthesis, Glycine, serine, and threonine metabolism, Glycerophospholipid metabolism, Linoleic acid metabolism, and Sphingolipid metabolism (Fig. 7E). By day 30, lipid metabolites, including phosphatidylcholine (PC), phosphatidylethanolamine (PE), and lysophosphatidylcholine (LysoPC), were significantly enriched in the A30Con group, while citric acid, proline betaine, alanyl − phenylalanine, 5 − hydroxy − L−tryptophan, L − acetylcarnitine, 3 − methylhistidine, and acetylcholine were significantly enriched in the A30Tre group (Fig. 7I; VIP > 1, *P* < 0.05). These metabolites were predominantly enriched in pathways related to Glycerophospholipid metabolism, D − Amino acid metabolism, Arginine and proline metabolism, Arginine biosynthesis, Alanine, aspartate, and glutamate metabolism, and Histidine metabolism (Fig. 7J).

## Discussion

Cattle of any age experience stress before and after transportation, with calves being particularly vulnerable during this process. This stress is multifactorial, notably exacerbated by the fasting and dehydration inherent to the transportation process, which increases the expenditure of functional energy and the risks of hunger and hypoglycemia, especially in young calves with limited body fat reserves [1]. The growth depression induced by transportation is one of the major challenges hindering the development of the cattle industry. Although there are some studies on the use of additives to alleviate transportation stress in calves [2, 9, 20], there is a lack of in-depth research on the effects of additives on calf growth performance and their underlying mechanisms. This situation may be attributed to several factors: the significant workload, the complexity of clinical and laboratory outcomes, and the challenge of managing already stressed calves. Our study evaluates the impact of CrPyr supplementation on the growth performance of calves transported over 3750 km and further explores its potential mechanisms using ruminal metagenome, metabolome, and serum metabolome approaches.

### CrPyr alleviates stress and improves growth performance of newly received cattle

Transportation has a significant detrimental effect on the growth performance of calves. Adult cattle may experience growth suppression for up to 28 days following transportation [21], and their immune function is also compromised [22]. The activation of immune Function requires the provision of 10–30% of the total energy from the body, with some estimates even reaching as high as 55% compared to the metabolizable energy typically required [23, 24]. Increasing dietary nutrient levels is a common strategy to provide additional energy to enhance the defense response. While a high-concentration diet may meet the energy demands for stress mitigation and immune system activation, thereby improving daily weight gain in newly received cattle, it can also exacerbate the imbalance in rumen bacterial flora homeostasis [5]. Therefore, administering nutritional supplements to newly received cattle is the optimal choice for optimizing their nutritional status and enhancing feed efficiency. CrPyr contains pyruvate and creatine, both of which are key energy substrates and critical antioxidants. Our published data indicated that CrPyr could improve ruminal microbial protein synthesis and immune function in heat-stressed beef cattle [17, 18], and aid in restoring rumen microbiota balance in finishing cattle experiencing transport stress [19]. In our studies, it was observed that the calves belonging to the CrPyr group exhibited a greater ADG compared with the Control group (Fig. 1C). This suggests that CrPyr has potential in enhancing the growth performance of newly received cattle. the specific mechanisms underlying its effects require further investigation.

The mechanisms underlying the growth-promoting effects of CrPyr in newly received cattle are multifaceted and likely involve modulation of stress levels, antioxidant capacity, and immune status, all of which are interrelated and collectively influence animal growth performance [25]. Elevated levels of COR and ACTH are known to negatively impact feed utilization efficiency in cattle, with higher levels of these hormones typically associated with reduced feed efficiency [26]. Previous studies have demonstrated that ACTH and COR levels significantly increase immediately after long-distance transport (14 h, 1000 km) and gradually return to normal levels approximately two weeks after arrival [27]. In the present study, CrPyr treatment significantly reduced serum ACTH and COR levels on day 1 after arrival, although no significant differences were observed on day 30 (Fig. 2A, B). These findings are consistent with our previous research, which showed that dietary supplementation with rumen-protected CrPyr could effectively lower serum COR concentrations in finishing beef cattle following transport [19]. The active components of CrPyr, such as creatine, are likely to contribute to these effects. Creatine monohydrate, which shares similar chemical properties with creatine, has been reported to act as a COR blocker and can reduce COR levels [28]. Therefore, the reduction in COR and ACTH levels observed in CrPyr-treated cattle suggests that CrPyr may mitigate stress responses associated with transportation.

Moreover, oxidative stress can disrupt the normal functioning of the immune system, diminish nutrient absorption and metabolism, and consequently impair growth performance [29]. In this study, the cattle in CrPyr group exhibited higher T-AOC, increased activities of SOD and GSH-Px, along with lower MDA concentration on days 1 and 30 post-transport (Fig. 2I, L). These findings indicated that CrPyr reduced oxidative stress in newly received cattle, which corresponded to the downregulation of COR and ACTH. The antioxidant capacity properties of CrPyr are partly attributed to its composition. Creatine has antioxidant properties protecting cells against superoxide anion and peroxynitrite [30]. Pyruvate can direct inhibition H_2_O_2_ through a non-enzyme catalyzed decarboxylation reaction [31]; or via the TCA, increasing the content of reducing coenzyme II through the pentose phosphate pathway. This indirect process consequently enhances the capability of the glutathione antioxidant system [32]. In addition, oxidative stress is usually accompanied by rapid inflammation. We also observed that the trends in pro-inflammatory cytokines (IL-1β, IL-6, and TNF-α) were consistent with MDA concentration in the CrPyr group (Fig. 2E, H). Therefore, we speculated that CrPyr can reduce the stress level and inflammatory response by alleviating the oxidative stress to promote the growth performance of newly received cattle.

### CrPyr changes the composition and function of rumen microbial of newly received cattle

Transportation stress is known to significantly alter the rumen microbiota composition, with a notable reduction in the abundance of dominant bacterial genera such as *Prevotella* and *Ruminococcus* [16, 33, 34]. *Ruminococcus*, as a beneficial gut bacterium, has been associated with diseases related to oxidative stress [35, 36]. In dairy cows with low oxidative stress, the abundance of *Ruminococcus* was significantly higher, and its role was linked to the upregulation of genes involved in glutathione (GSH) synthesis [37]. In our study, we observed a significant increase in the abundance of *Ruminococcus* species, including *R. bacterium_P7*, *R. sp.*, *R. bromi*, *R. albus*, *R. bacterium_FB2012*, and *R. sp._JE7A12*, in the CrPyr-treated group on day 4 post-transport (Fig. 3A, C). This indicates that CrPyr has a substantial impact on the recovery of *Ruminococcus*. Moreover, our correlation analysis revealed a significant positive relationship between the altered *Ruminococcus* species and serum antioxidant indicators (SOD, GSH-Px, and T-AOC) (Fig. S3A). This suggests that CrPyr may mitigate oxidative stress in newly received cattle by enhancing the abundance of *Ruminococcus*, which in turn supports the antioxidant system. This finding provides additional evidence that CrPyr can alleviate oxidative stress through modulating the rumen microbiota. On the other hand, it has been reported that *Ruminococcaceae* bacteria are primarily involved in nitrogen and amino acid metabolism [38, 39]. Interestingly, during the same period, 6 significantly upregulated KEGG metabolic pathways were related to amino acid metabolism (Fig. 4A), and these pathways were positively correlated with the abundance of *Ruminococcus* (Fig. S3A). This result is consistent with the observed increase in rumen NH₃-N content during this period (Fig. 4C). For several decades, creatine has historically served as a nitrogen supplement in ruminant nutrition [40]. These create favorable conditions for the synthesis of microbial crude protein (MCP). Earlier studies have indicated that supplementing CrPyr under stress conditions can notably enhance the synthesis of ruminal MCP in beef cattle [17–19]. This dual effect of CrPyr—enhancing the abundance of *Ruminococcus* and promoting nitrogen metabolism—likely contributes to the overall improvement of rumen Function and health in transported calves. On day 30 post-transport, the bacterial composition underwent significant changes. During this period, in the CrPyr-treated group, 12 of the 25 significantly upregulated bacterial species belonged to the genus *Prevotella* (Fig. 3D), indicating that the regulatory effects of CrPyr on newly received cattle are sustained. *Prevotella* species utilize starch and protein to produce succinate and acetate and are among the core species in the rumen of ruminants [41]. Xue et al. [42]. have demonstrated that *Prevotella* plays a crucial role in the synthesis of VFAs. Consistent with this, the concentrations of acetate and butyrate were significantly higher in the CrPyr-treated group on day 30 (Fig. 4C), suggesting that CrPyr can enhance energy supply to the host during this period.

SLP and ETP are the primary pathways for microbial ATP synthesis and utilization [43]. Under anaerobic conditions, most microbial fermentations primarily produce ATP via SLP, which promotes ATP synthesis through glycolysis, with high-energy phosphate groups being directly transferred from substrate molecules to ADP [44]. Meanwhile, ETP utilizes the electrochemical potential generated by the respiratory chain to drive efficient ATP synthesis via ATP synthase (also known as ATPase) [45]. ETP is facilitated by three rotary ATPases responsible for this fundamental energy conversion: reversible protein complexes comprising eukaryotic vacuolar H^+^-ATPases (V-ATPase), bacterial or archaeal V/A ATPases, and F-type F₀F₁ ATPases [46]. In this study, the abundance of SLP was higher in the CrPyr-treated group on both day 4 and day 30. At the same time, the ETP pathways, specifically the V/A type and F-type, were significantly higher in the CrPyr-treated group on day 4 and day 30, respectively (Fig. 5A, C). Further analysis of differentially abundant genes revealed that the abundance of *ATPVC* on day 4 and *ATPF₀A* on day 30 was higher in CrPyr group (Fig. 5B, D). This suggests that CrPyr promotes ATP synthesis modulating the ETP pathway, thereby supporting the proliferation of beneficial bacteria such as *Ruminococcus* and *Prevotella*. This mechanism aligns with previous study showing that ATP availability is crucial for microbial growth and rumen function [47].

### CrPyr regulates rumen and serum purine metabolism and amino acid metabolism to alleviate stress in newly received cattle

Rumen metabolomics supported microbial Functional genomic interpretations, while serum metabolomics can reveal the interaction mechanisms between the rumen host and microbes and their metabolites. On day 4, the differential rumen metabolites were primarily associated with purine metabolism, including Xanthosine, 7-Methylinosine, and Guanosine, which were significantly elevated in the CrPyr group (Fig. 6D). This indicates robust nitrogen metabolism, likely increasing ammonia availability and thereby promoting the synthesis of purine nucleotides. This metabolic shift is crucial for microbial growth, as purine nucleotides are essential for DNA and RNA synthesis, as well as for generating energy-rich molecules like ATP [48]. These findings are consistent with the results of KEGG pathway analysis and changes in ATP content during this period. Guanidinoacetic acid (GAA), a crucial synthetic precursor of creatine, was decreased in the ATre group on day 4 post-transport. GAA is vital for energy supply, particularly in newly received cattle that often experience substantial stress due to fasting and water deprivation, resulting in an inadequate energy supply. CrPyr supplementation may enhance the conversion of GAA to creatine, thereby improving the energy status of the rumen ecosystem [49, 50]. This enhanced energy availability not only supports microbial growth but also helps the host animal withstand stressors associated with transport and fasting. During this period, the serum metabolites that showed significant differences were mainly enriched in Arginine biosynthesis and Glycine, serine, and threonine metabolism (Fig. 7E), indicating that host nitrogen metabolism was also enhanced at this time. Specifically, the relative abundance of serum aspartate significantly increased during this period. Aspartate has been established to possess anti-inflammatory and antioxidant properties [51]. Jin et al. [52] found that under oxidative stress conditions, the supplementation of aspartate could promote the proliferation of *Ruminococcaceae* and regulate the antioxidant and inflammatory signaling pathways by modulating mitochondrial function, enhancing the expression of antioxidant-related genes, and inhibiting the activation of inflammatory factors, thereby maintaining host health. The results of the present study align with these findings, and correlation analysis also indicates a positive relationship between *Ruminococcaceae* and aspartate levels (Fig. S3B). This suggests that CrPyr may enhance the host antioxidant capacity by promoting the generation of aspartate, which could be a significant reason for the alleviation of oxidative stress during this period. However, the specific mechanisms by which CrPyr promotes the production of aspartate in the host, as well as the detailed molecular pathways through which aspartate alleviates oxidative stress in stressed calves, remain to be further investigated.

**Fig. 8:**
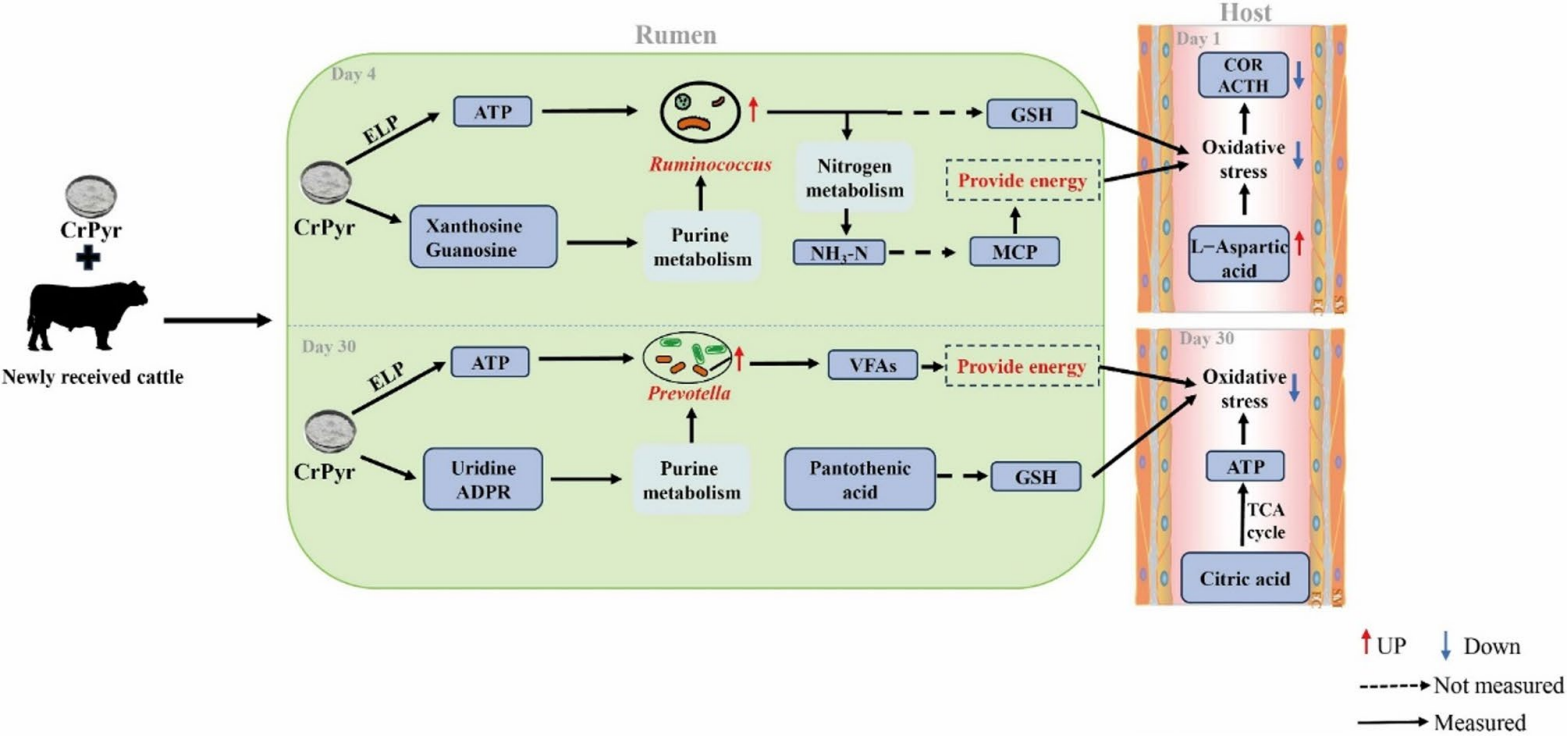
Schematic diagram of CrPyr alleviating stress in newly introduced cattle

The identified ruminal differential metabolites in the A30Tre vs. A30Con comparison shed light on the significant alterations in key metabolites such as adenosine diphosphate ribose (ADPR), uridine, N-Acetyl-L-glutamic acid, and pantothenic acid, all of which exhibited upregulation in the A30Tre group (Fig. 6I). These differential metabolites were primarily enriched in the pathways of Arginine and proline metabolism and Purine metabolism (Fig. 6E). ADPR plays a crucial role in DNA repair, while uridine is a major precursor for RNA synthesis. The increased abundance of both ADPR and uridine may provide a foundation for the proliferation of *Prevotella* during this period by supplying essential nucleotides for bacterial DNA and RNA synthesis, as well as supporting the energy metabolism required for microbial growth. Pantothenic acid, as an important precursor for the synthesis of coenzyme A, participates in various reactions in the body, including the elevation of GSH levels to alleviate oxidative stress [53]. The increase in pantothenic acid levels may represent another pathway by which CrPyr mitigates host oxidative stress, although the specific mechanisms underlying this increase require further investigation. During this period, serum metabolite analysis revealed a significant increase in citric acid levels (Fig. 7I), which suggested that CrPyr might enhance the activity of the serum TCA cycle to provide energy for newly received cattle on day 30 post-transport. As is well known, the TCA cycle is recognized as the primary metabolic pathway for energy generation, primarily stemming from the metabolism of carbohydrates and fatty acids. Previous research has associated certain processes within the TCA cycle pathway with stress response [54, 55]. The elevated serum ATP content observed during this period may be related to this finding (Fig. 2M). Concurrently, lipid compounds (PC, phosphatidylethanolamine PE, and LysoPC) were significantly reduced in the treatment group. A previous study has emphasized that a higher content of PC and lysoPC could enhance the antioxidant capacity of newly received cattle. A previous study has emphasized that a higher content of PC and lysoPC could enhance the antioxidant capacity of newly received cattle [34]. Dietary supplementation with PC reportedly improved brain function and fostered oxidative stress resistance by modulating the activity of SOD in mice [56]. These studies collectively indicated the positive antioxidant effects of PC. However, we found a paradoxical decrease in PC content despite the increase in antioxidant capacity. This phenomenon may be attributed to CrPyr promoting the transport of PC to the rumen epithelial cells, rather than inhibiting its synthesis, thereby affecting PC levels. As a key component of the rumen mucosal barrier, the enrichment of PC may enhance intestinal barrier function, reduce the translocation of endotoxins into the bloodstream, and thus indirectly attenuate inflammation [57] (Fig. 8).

## Conclusion

Our study demonstrates that CrPyr significantly alleviates transportation stress in calves, enhances their antioxidative capacity, and promotes growth, with mechanisms that are time-dependent (Fig. 8). Within the first 4 days post-transport, CrPyr modulates ruminal nitrogen metabolism by increasing *Ruminococcus* abundance and regulating ATP synthesis genes, which supports microbial protein synthesis and boosts the host’s antioxidative capacity. At day 30 post-transport, CrPyr primarily regulates the synthesis of ruminal VFAs by increasing *Prevotella* abundance and regulating ATP synthesis genes, providing energy for the host’s growth. These findings suggest that CrPyr is an effective nutritional strategy to mitigate transportation stress and promote growth in newly received cattle, with significant implications for the beef cattle industry.

## Methods

### Animal treatments and experimental design

The administrator of beef cattle company purchased 100 Simmental crossbred calves, approximately six months old, in excellent health (without any signs of illness) from the Live Animals and Forage Trading Market in Altay city, Xinjiang Province. Each of the 100 calves was randomly assigned a unique number from 1 to 100. Subsequently, they were randomly divided into two adjoining pens, namely pen I and pen II. Pen I and II accommodated 50 calves, respectively. The objective of this configuration was to oversee their behavior and facilitate their mingling for three consecutive days. Before transport, the ratio of concentrate and wheat straw in diet was 30:70. The concentrate was purchased from commercial company, including crude protein ≥ 17%, crude fiber ≤ 10%, crude ash ≤ 9%, moisture ≤ 14%, total phosphorus ≥ 0.4%, calcium 0.5–1.2%, salt 0.8–1.5%. Once the commingling was complete, the all calves were transported by the double layer car, length 13.5 m, width 2.3 m and height 4.2 m (manufactured by Zhumadian CIMC HuaJun Vehicle Co., Ltd, China). The vehicle was equipped with quilt mattress and covering. The journey commenced at 20:00 h on September 13, 2021, transporting cattle from the Live Animals and Forage Trading Market in Altay city, Xinjiang Province, to Yuzhou city, Henan Province. The transport arrived at Yuzhou Longyue Animal Husbandry Co., Ltd, Xuchang, Henan, at 15:00 h on September 16, 2021. The total travel time was 67 h, covering a distance of 3450 km on highways and city roads at a maximum speed of 70 km/h.

The day before transportation, CrPyr was provided to the calves in pen II at a dose of 30 g/day and mixed with their drinking water. This dosage was referenced from Liu [18]. During transportation, cattle were deprived water but had access to some wheat straw, which was placed on the sides of the cart in the form of straw bundles when loading. After arrival, the CrPyr treatment calves were tethered in the same barn. Nine calves were randomly selected as the experimental group (CrPyr group), while eight calves were randomly selected from pen I as the control group (Control group). The selection of the number of experimental animals was based on cost considerations. The experimental group continued to receive 30 g/day of CrPyr in their drinking water for 30 days, while the control group received no treatment. Initially, for the first two days, cattle were predominantly fed with wheat straw. Subsequently, cattle were transitioned to a total mixed ration (TMR). Table S1 illustrates the composition and nutrient levels of the TMR, which was provided twice daily. Drinking water intake of beef cattle was strictly controlled in the first five days after arrival. The water temperature was about 37 °C, given three times a day with about 3 L each time, and in subsequent experiments, normal temperature water was available freely.

### Sample collection and measurements

#### Determination of the growth performance

On the days 1 and 30 after arrival, we measured the body weights at 09:00 of the newly received cattle following a 24-hour fasting period. We also recorded the daily feed intake of these animals on the last three days of every ten-day period (specifically on days 8, 9, 10; 18, 19, 20; and 27, 28, 29). From this data, we calculated the average daily feed intake (ADFI), the average daily gain in weight (ADG), and the ratio between the quantity of feed consumed and the weight gained (F: G).

#### Blood and rumen fluid collection

The collection of rumen fluid and blood was conducted on fully conscious and unanesthetized cattle. Initially, the cattle were appropriately restrained to ensure their safety and facilitate the smooth execution of the experimental procedures. Restraint is a common animal management technique used to limit movement for various handling purposes. While restrained, rumen fluid and blood were collected. During receiving period, blood and rumen fluid samples were collected on days 1, 4, and 30. Specifically, blood samples were drawn from the jugular vein of the cattle into EDTA vacutainer tubes on days 1 and 30. The samples were centrifuged at 3000×g for 15 min at 4 °C to collect serum. Concurrently, to cooperate with the management of the cattle farm, rumen contents were sampled using oral stomach on days 4 and 30 after transport. Between each collection, the device was rigorously cleaned with running water, and the initial 50 mL of rumen fluid was discarded to reduce saliva contamination. Subsequently, 50 mL of the rumen fluid sample from each cattle was immediately assessed for pH using a portable pH meter (HANNA Instruments, Cluj-Napoca, Romania). After that, both the serum and rumen fluid were promptly frozen in liquid nitrogen and stored at − 80 °C for subsequent analysis. The newly received cattle in control and CrPyr group were divided into 4 subgroups based on the sampling time: ACon and ATre = day 1 or 4 after transport, collection of blood on the day 1 and rumen fluid on the day 4; A30Con and A30Tre = day 30 after transport, simultaneous collection of blood and rumen fluid.

#### Chemical analyses

The concentrations of serum, including cortisol (COR), adrenocorticotropic hormone (ACTH), total antioxidant capacity (T-AOC), superoxide dismutase (SOD), glutathione peroxidase (GSH-PX), malondialdehyde (MDA), IgA, IgG, IgM, interleukin-1β (IL-1β), IL-4, IL-6, tumour necrosis factor-α (TNF-α), ATP and pyruvate were measured using commercial assay kits from Nanjing Jiancheng Bioengineering Institute (Nanjing, China) according to the manufacturer’s instructions. The VFA concentrations in the rumen fluid samples were determined using gas chromatography (Shimadzu GC-2014, Japan) equipped with a capillary column (Stabilwax, Restek, Bellefonte, PA, USA). The concentration of NH_3_-N was determined using alkaline sodium hypochlorite-phenol spectrophotometry.

### Rumen metagenomics

#### DNA extraction, library construction, and metagenomic sequencing

The metagenomic DNA extraction, library construction, and metagenomic sequencing were same as previous method [34]. Total genomic DNA was extracted from rumen fluid samples in both the control and CrPyr groups at two time points (*n* = 6 for each group) by using the FastDNATM Spin Kit for Soil (MP Biomedicals, USA). The concentration and purity of the extracted DNA were subsequently evaluated using the TBS-380 fluorometer and NanoDrop2000 spectrophotometer, respectively. DNA extract was fragmented to an average size of about 400 bp using Covaris M220 (Gene Company Limited, China) for paired-end library construction. Paired-end sequencing was performed on Illumina NovaSeq 6000 (Illumina Inc., San Diego, CA, USA) at Majorbio Bio-Pharm Technology Co., Ltd. (Shanghai, China). Sequence data associated with this project have been deposited in the NCBI Short Read Archive database (BioProject ID: PRJNA943223).

The raw metagenomic sequencing data were processed to obtain high-quality clean reads. This was achieved by filtering out low-quality reads (quality scores < 20 or length < 50 bp or having N bases) using the fastp tool [58] (version 0.20.0, https://github.com/OpenGene/fastp) on the free online platform of Majorbio Cloud Platform (cloud.majorbio.com). Host reads were aligned the bovine genome using BWA (version 0.7.9a, http://bio-bwa.sourceforge.net) to host DNA [59]. The filtered reads were de novo assembled for each sample using Megahit [60]. MetaGene [61] (http://metagene.cb.k.u-tokyo.ac.jp/) was used to predict open reading frames (ORFs) from the assembled contigs with the length > 100 bp. Non-redundant contigs were identified using CD-HIT [62] (version 4.6.1, http://www.bioinformatics.org/cd-hit/) with 90% sequence identity and 90% coverage. The quality-filtered sequence reads were mapped to the representative sequences with 95% identity using SOAPaligne [63] (version 2.21, http://soap.genomics.org.cn/). and the gene abundance in each sample was calculated as reads per kilobase per million mapped reads (RPKM).

Representative sequences of non-redundant gene catalog were aligned to the NCBI NR database using Diamond [64] (version 0.8.35, http://www.diamondsearch.org/index.php) for taxonomic annotations using the blastp (version 2.2.28+, http://blast.ncbi.nlm.nih.gov/Blast.cgi). Feature abundance was normalized using the relative abundance of each sample. Microbial taxa with average relative abundances > 0.01% were used for downstream analysis. The KEGG annotation was conducted using Diamond [64] against the Kyoto Encyclopedia of Genes and Genomes database (version 94.2, http://www.genome.jp/keeg/) and RPKM with average relative abundances > 100 were used for downstream analysis. All these databases had an E-value cut- of 1e^−5^ while annotating ORFs.

#### Rumen and serum metabolomics

The rumen and serum samples were analyzed using the LC-MS platform (Thermo, UHPLC -Q Exactive HF-X).

#### Metabolite extraction

The extraction of metabolites from rumen fluid and serum was conducted following previously method [17].

#### Metabolomics data analysis

The LC-MS data were processed using Progenesis QI 2.3 (Waters Corporation, Milford, USA) to extract raw peaks, filter and calibrate baseline, align peaks, deconvolute, identify peaks, and integrate peak areas. Rumen and serum metabolite that were present in < 50% of samples or with a relative standard deviation (RSD) > 30% were removed. Following normalization procedures and imputation, statistical analysis was performed on log transformed data to identify significant differences in metabolite levels between comparable groups. The analysis of metabolite sources was performed in MetOrigin (2024-07 version; https://metorigin.met-bioinformatics.cn) [65]. A multivariate statistical analysis was performed using ropls (Version1.6.2, http://bioconductor.org/packages/release/bioc/html/ropls.html) R package from Bioconductor on Majorbio Cloud Platform (https://cloud.majorbio.com). The partial least squares discriminate analysis (PLS-DA) and *t* test were performed between the Control and CrPyr groups, with *P* value < 0.05 and the VIP > 1 being considered as significantly different metabolites. The enrichment analysis included in MetOrigin was applied to each metabolite from each cluster to identify metabolic pathways (*P* < 0.05) [65].

### Statistical analysis

Growth performance, serum biochemical parameters and rumen fermentation parameters were compared using *t* test with SPSS (version 17.0, IBM, Armonk, NY, USA). Rumen microbiome and function were compared using Wilcoxon rank-sum test, *P* value < 0.05 being considered as significantly different. Correlation analysis between rumen bacteria, serum indicators, rumen metabolome, and serum metabolome was performed using Spearman’s rank correlation and visualized in a heatmap format using R (Version 3.3.1, R Core Team, Vienna, Austria) “pheatmap package”. The absolute value of correlation coefficients (|r|>0.50 and *P* < 0.05) was considered as significant.

## Supplementary Information

Supplementary material 1.

Supplementary material 2.

## Data Availability

The datasets analysed during the current study are available from the corresponding author on reasonable request. Sequence data associated with this project have been deposited in the NCBI Short Read Archive database (BioProject ID: PRJNA943223).

## Abbreviations

ACTH: Adreno cortical tropic hormone
CAZymes: Carbohydrate-active enzymes
CK: Creatine kinase
COR: Cortisol
GSH-PX: Glutathione per-oxidase
IL-1β: Interleukin-1β
KEGG: Kyoto encyclopedia of genes and genomes
MCP: Microbial crude protein
MDA: Malondialdehyde
PC: Phosphatidylcholine
SOD: Uperoxide dismutase
T-AOC: Total antioxidant capacity
TNF-α: Tumor necrosis factor alpha
VFA: Volatile fatty acid
VIP: Variable importance in the projeciton
RA: Relative abundance
