# Supplementary material for: Influence of creatine pyruvate on newly received cattle: insights from metagenomics and metabolomics
Source: BMC Microbiol. 2025 Oct 10;25:658. doi: 10.1186/s12866-025-04384-8 (PMC12512676; doi:10.1186/s12866-025-04384-8)
Supplement: Supplementary file 1 — Supplementary material 1. [file 12866_2025_4384_MOESM1_ESM.docx]

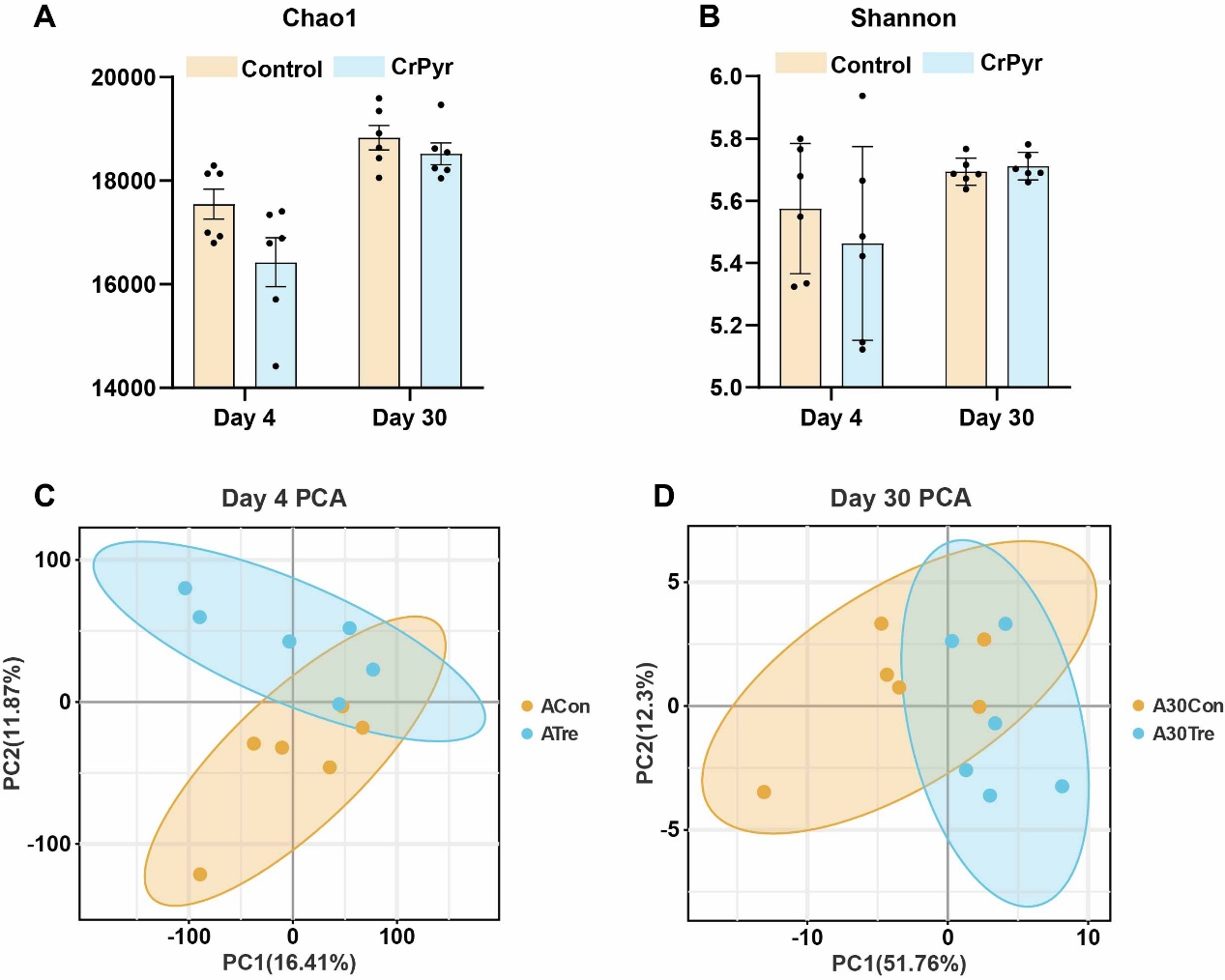


Fig. S1 Microbial compositional profiles of newly received cattle at days 4 and 30 after transport. **A-B** Bacteria alpha diversity between the control and CrPyr groups. **C-D** Rumen bacteria compositional profiles of the Control and CrPyr groups based on species visualized using PCA. day 4 = at day 4 after transport; day 30 = at day 30 after transport.


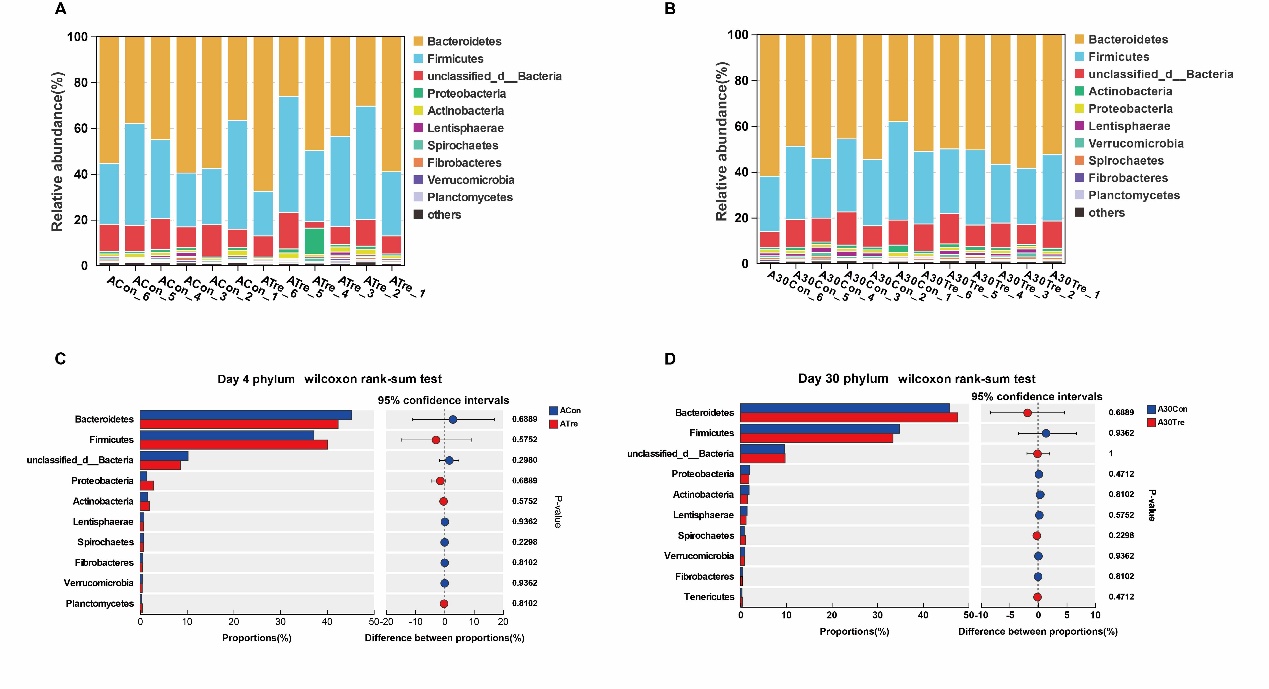
 Fig. S2 Differential rumen bacteria phylum of newly received cattle between the control and CrPyr groups on days 4 and 30 after transport. **A, B** the composition of rumen bacteria phylum on day 4 and 30. **C, D** Significantly differential bacterial at phylum on day 4 and 30 after transport. Significant differences at phylum were tested by Wilcoxon rank-sum test.


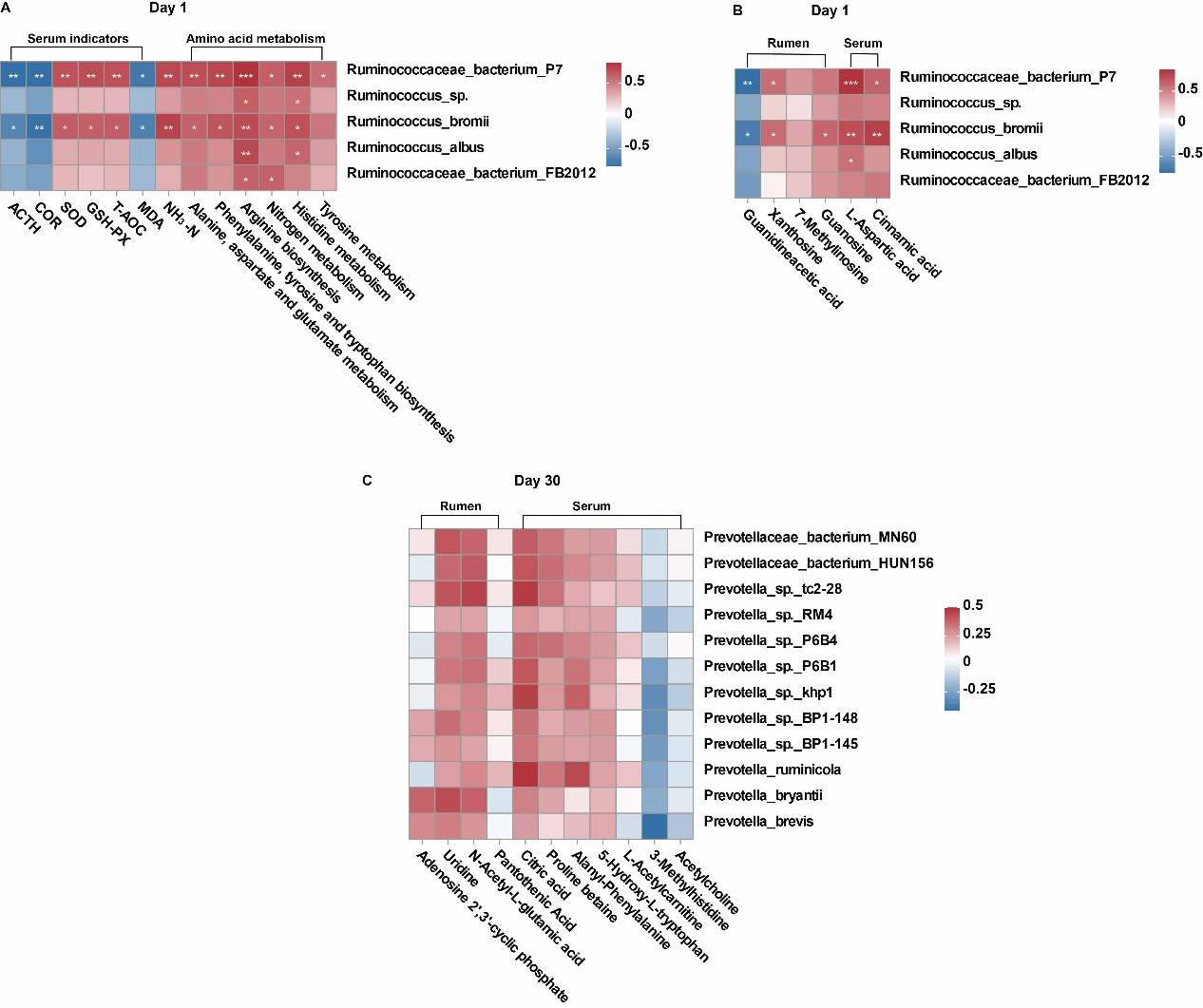


Fig. S3 Heatmap showing the association between ruminal significant differential bacterial species and serum hormone, antioxidant indicators, rumen and serum metabolites. **A** differential bacterial species and serum hormone, antioxidant indicators and amino acid metabolism pathway on day 1. **B** differential bacterial species and rumen and serum metabolites on day 1. **C** differential bacterial species and rumen and serum metabolites on day 4 (Spearman’s correlation, |R| > 0.5, *P* <0.05).
